# The Tetrahydrofuran Motif in Marine Lipids and Terpenes

**DOI:** 10.3390/md20100642

**Published:** 2022-10-15

**Authors:** Paula González-Andrés, Laura Fernández-Peña, Carlos Díez-Poza, Asunción Barbero

**Affiliations:** 1 Department of Organic Chemistry, Campus Miguel Delibes, University of Valladolid, 47011 Valladolid, Spain; 2 Departamento de Química Orgánica y Química Inorgánica, Facultad de Farmacia e Instituto de Investigación Química Andrés M. del Río (IQAR), Universidad de Alcalá, Ctra. Madrid-Barcelona, 28871 Madrid, Spain

**Keywords:** marine natural products, oxygen heterocycles, tetrahydrofuran, total synthesis, biological activity, terpenes, fatty acids

## Abstract

Heterocycles are particularly common moieties within marine natural products. Specifically, tetrahydrofuranyl rings are present in a variety of compounds which present complex structures and interesting biological activities. Focusing on terpenoids, a high number of tetrahydrofuran-containing metabolites have been isolated during the last decades. They show promising biological activities, making them potential leads for novel antibiotics, antikinetoplastid drugs, amoebicidal substances, or anticancer drugs. Thus, they have attracted the attention of the synthetics community and numerous approaches to their total syntheses have appeared. Here, we offer the reader an overview of marine-derived terpenoids and related compounds, their isolation, structure determination, and a special focus on their total syntheses and biological profiles.

## 1. Introduction

Marine organisms are a source of intriguing and fascinating compounds. These living beings have continuously evolved over time, since they are part of the oldest habitat on earth. Being also the largest ecosystem, the ocean has the potential to offer innumerable compounds with interesting biological activities yet to be discovered [1]. This is supported by the fact that hundreds of new molecules are reported within the scientific community every year [2,3,4].

Usually, the isolation of pure active compounds is a time-consuming and expensive process, due to the need of efficient extraction processes and sequential purification steps. Moreover, large amounts of raw materials have to be collected to finally isolate fairly low quantities of the desired compounds.

Fortunately, the great contribution of chemists in the field of total synthesis and asymmetric catalysis over the last decades has had countless benefits. On one hand, even though nuclear magnetic resonance (NMR) techniques are very powerful tools, in some cases, the characterization of complex molecules can be difficult, leading to misassignments [5]. Fortunately, total synthesis has emerged as a—somewhat—costly but effective tool for the determination of the absolute configuration of marine metabolites. On the other hand, synthesis provides access to sufficient quantities of the desired compounds for further extensive biological studies.

Within the marine-derived metabolites, terpenes represent one of the most significant families. They are a large and diverse group of compounds that usually present valuable pharmacological properties. Various reviews summarize the discovery of a high number of these metabolites in recent years from different sources, namely sponges [6,7], fungi [8,9,10], and corals [11,12], among others [13,14].

Other common structural motifs present in marine drugs are heterocycles. Within them, five-, six-, and seven-membered oxygenated heterocycles are frequently found in such bioactive compounds. The six-membered tetrahydropyrans, the most abundant, are common targets of study [15,16]. The corresponding seven-membered oxepanes, and their appearance in relevant bioactive marine compounds, were reviewed by our group [17]. Regarding tetrahydrofurans, Fernandes and coworkers recently reviewed the most iconic examples of total synthesis of 2,3,5-trisubstituted tetrahydrofuran-containing natural products [18,19]. We have also recently summarized the synthesis and biological properties of marine-derived tetrahydrofuran-containing compounds, focusing on the polyketide family [20].

**Figure 1:**
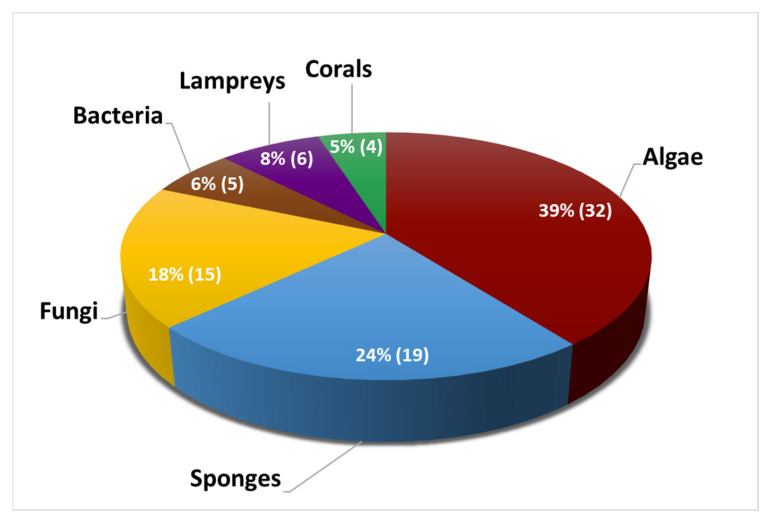
Distribution of the number of metabolites from this review, according to their marine source (color code coincides with that of Table 1).

**Figure 2:**
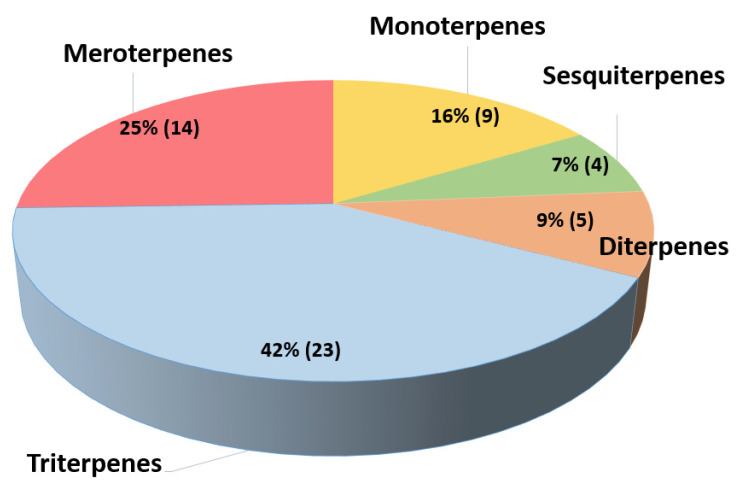
Distribution of compounds from this review according to terpenoid type.

Continuing our series, here we give an overview of tetrahydrofuran-containing marine drugs, focusing on the terpene family and related compounds. We searched SciFinder for tetrahydrofuran-containing compounds with biological activity, focusing on the period 2000–2022. Our search was refined to compounds of the terpenoid family of compounds of marine origin, finding 81 compounds (Table 1). The main source (see Figure 1) was algae (32 compounds), followed by sponges (19 compounds), fungi (15 compounds), lampreys (6), bacteria (5), and corals (4). Excluding nonterpenoid lipids, we found 55 terpenoid compounds (see Figure 2), most of them being triterpenes (23) followed by meroterpenes (14), monoterpenes (9), diterpenes (5) and, finally, sesquiterpenes (4). 

We want to offer the reader an overview of the most recent tetrahydrofuran-containing terpenoids and related compounds of marine origin, their natural source of isolation, biological properties, and synthetic strategies towards them. We have also included classic examples, as their structure and activity are closely related to the more recently isolated metabolites and help to understand structure–activity relationships. We start with some examples of fatty acids with interesting biological profiles, and then move to the broad family of terpenoid compounds (monoterpenes, sesquiterpenes, diterpenes, triterpenes, and meroterpenoids). At the end, we also highlight other small THF-containing compounds that show interesting biological activities or were recently isolated and, thus, have potential to be shown in the near future.

## 2. Lipids

### 2.1. Lipid Alcohols

#### C19 Lipid Diols

**Figure 3:**
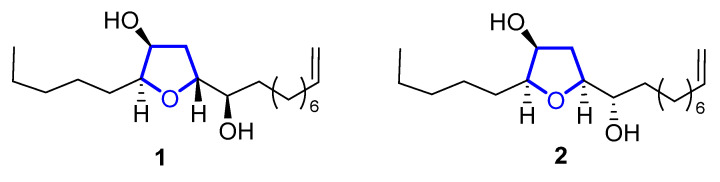
Structure of *trans*-oxylipid **1** and *cis*-oxylipid **2**.

Diastereomeric *trans*-oxylipids **1**, and *cis*-oxylipid **2** (Figure 3), were isolated in 1980 [21] and 1998 [22], respectively, from the brown alga *Notheia anomala*. Their structure and relative stereochemistry were assigned by 1D and 2D NMR spectral data and confirmed by single-crystal X-ray analysis. Their absolute configuration was determined by the Horeau method in the case of compound **1**, and by the advanced Mosher method for compound **2**. Both of them display in vitro antihelmintic activity, inhibiting larval development in parasitic nematodes. The *trans*-isomer **1** showed LD_50_ values against *Haemonchus contortus* (1.8 ppm) and *Trichostrongylus colubriformis* (9.9 ppm), comparable to those of the commercial nematocides levamisole and closantel. Synthetic routes for these oxylipids were recently reviewed [18].

**Scheme 1:**
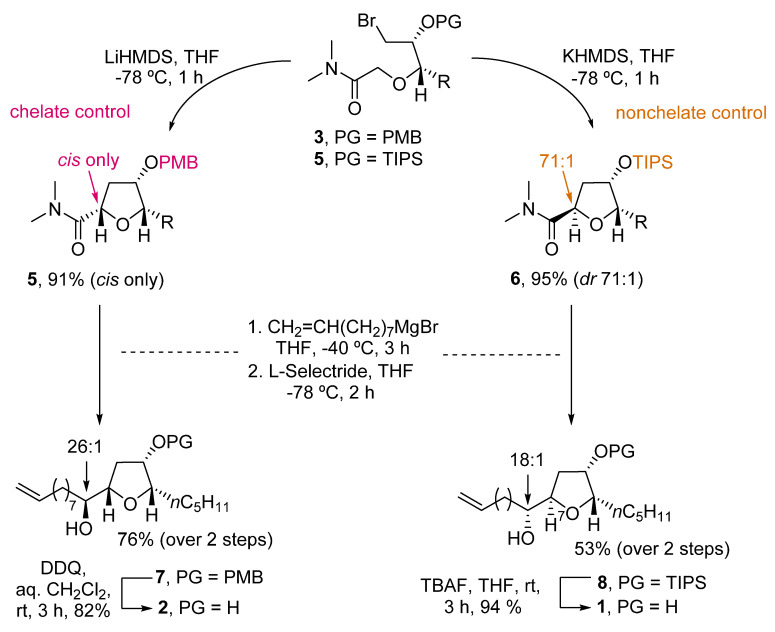
Stereodivergent synthesis of *trans-* and *cis*-oxylipids **1** and **2** by Kim.

An elegant example of the stereodivergent synthesis of both isomers was developed by Kim et al. [23]. They developed an intramolecular amide enolate alkylation, where the C3-hydroxy protecting group selection permits the formation of the desired isomer (Scheme 1). Thus, starting from PMB-protected bromoamide **3**, reaction with LiHMDS afforded only the *cis*-product **4**. The preferent formation of the *cis*-isomer was due to the chelating ability of the PMB group. Therefore, when using a nonchelating group such as TIPS (compound **5**), the reaction with KHMDS predominantly yielded the *trans*-THF **6**. Further reaction of **4** and **6** with CH_2_=CH(CH_2_)_7_MgBr and reduction with L-selectride afforded **7** and **8** in good yields (76% and 53% over two steps, respectively). Deprotection of **7** with DDQ, and **8** with TBAF, respectively, gave *cis*-oxylipid **2** in 82% yield and the *trans*-isomer **1** in 94% yield.

### 2.2. Fatty Acids

#### 2.2.1. Petromyroxols

**Figure 4:**
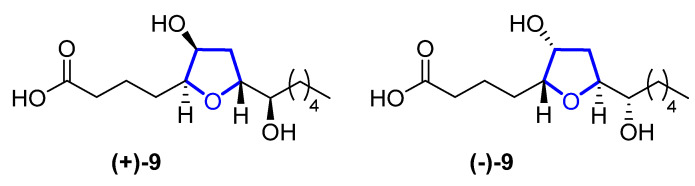
Structures of (+)-and (-)-petromyroxol **9**.

In 2015, Li reported the isolation of (+)- and (−)-petromyroxols (**9**) [24]. They are oxylipids isolated from water conditioned with larval sea lamprey *Petromyzon marinus* L. Interestingly, these molecules are the first tetrahydrofuran acetogenindiols isolated from a vertebrate animal (Figure 4). The absolute configuration of each enantiomer was determined by a combination of Mosher ester analysis and comparison with related natural and synthetic products. The **(+)-9** shows a potent olfactory response of 0.01 to 1 μM in the sea lamprey, while the (−)-isomer has a softer effect. Synthetic routes towards them were recently reviewed [18]. 

**Scheme 2:**
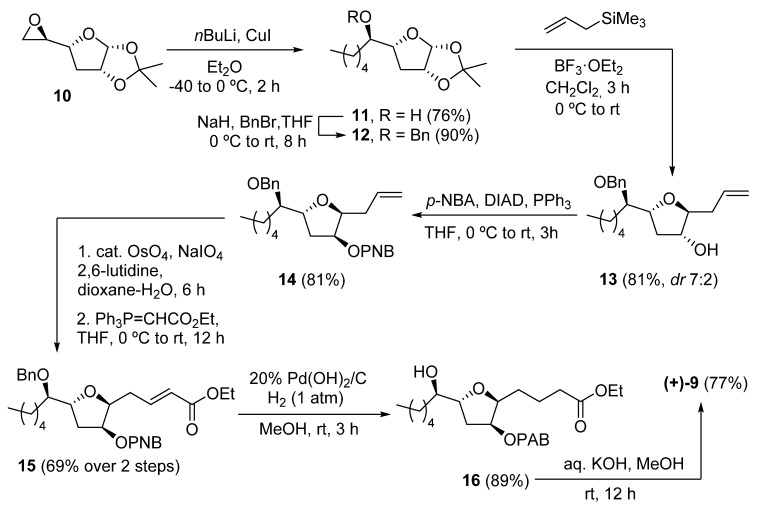
Synthesis of (+)-petromyroxol by Ramana and coworkers.

A recent example of the synthesis of **(+)-9**, along with all possible diastereoisomers, was presented in 2020 by Ramana and coworkers [25]. The synthetic route started from the commercial THF compound **10** (Scheme 2). The alkyl chain was installed by reaction with the appropriate cuprate. Subsequent benzyl protection of **11** afforded **12**, which after reaction with allyltrimethylsilane, yielded the desired diastereomer (7:2 ratio) of the allylated THF **13**. After protection with a para-nitrobenzoate (PNB) group under Mitsunobu conditions, compound **14** was subjected to oxidative olefin cleavage with OsO_4_/NaIO_4_ and subsequent Wittig olefination to obtain a-b-unsaturated ester **15**. Hydrogenation with Pearlman catalyst (20% Pd(OH)_2_/C) afforded **16** in 89% yield, where the benzyl group, the double bond, and the nitro group were all reduced. Finally, hydrolysis of both ester groups with KOH in methanol afforded the desired (+)-petromyroxol in 77% yield.

#### 2.2.2. PMA

**Figure 5:**
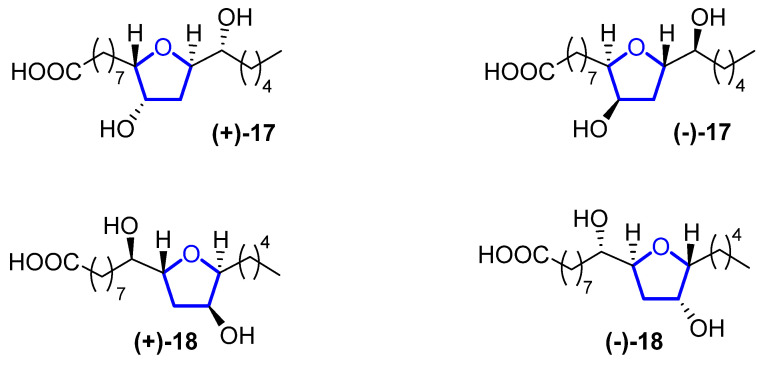
Structure of (+)-PMA (**(+)-17**), (−)-PMA (**(−)-17**), (+)-PMB (**(+)-18**), and (−)-PMB (**(−)-18**).

Petromyric acids A and B (PMA and PMB) are dehydroxylated tetrahydrofuranyl fatty acids that were isolated from larval washing extracts from the sea lamprey *Petromyzon marinus* in 2018 [26]. From the washing extract, four fatty acids related to the acetogenin family were identified: (+)-PMA (**(+)-17**), (−)-PMA (**(−)-17**), (+)-PMB (**(+)-18**), and (−)-PMB (**(−)-18**) (Figure 5). Their chemical structure was elucidated by NMR spectroscopy and confirmed by chemical synthesis and X-ray crystallography.

Sea lampreys are anadromous fishes that migrate, using their olfactory cues to orientate, from the ocean to freshwater to find a suitable spawning stream. When approaching river mouths, the decision of which stream is optimal to spawn in is taken using their olfactory system to detect a pheromone emitted from larval sea lampreys. When investigating larval washing extracts, four fatty acids were identified, but only **(+)-17** has proven to be the pheromone that guides lamprey adults. However, its enantiomer, **(−)-17**, does not produce the same behavioral effect. Fatty acid analogues have been reported to be pheromones in insects, but this is the first identification in fish. The sea lamprey is a destructive invader in the Laurentian Great Lakes, while in Europe, its population has decreased precipitously, so **(+)-17** can be used for the control and conservation of their populations.

Although they have a high potential for application, to the best of our knowledge, no total synthesis has been reported so far for these compounds.

#### 2.2.3. Mutafurans

**Figure 6:**
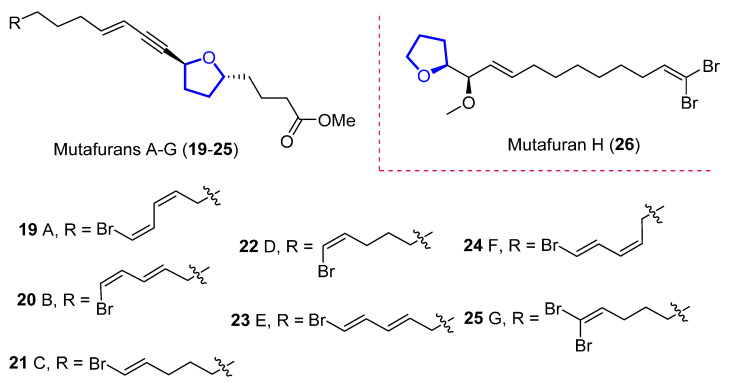
Structure of mutafurans A–G and the yne-lacking mutafuran H.

Mutafurans A–G (**19**–**25**) are brominated ene-ynetetrahydrofurans (Figure 6) that were isolated by Molinski in 2007 from the marine sponge *Xextospongia muta* [27]. Later, Liu reported the isolation of mutafuran H (**26**), a brominated ene-tetrahydrofuran isolated from sponge *Xextospongia testudinaria* within other sterols and brominated compounds [28]. Their structure and absolute configuration were determined by 1D and 2D magnetic resonance, mass spectrometry, and circular dichroism.

Mutafurans A–D showed moderate antifungal activity against the fungus *Cryptococcus neoformans var. grubii,* but were inactive against *Candida albicans* (ATCC14503 and 96–489) and *Candida glabrata* [27]. Furthermore, mutafuran H showed biological activity against *Artemia salina* larvae (LC_50_ = 2.6 μM) and a significant acetylcholinesterase inhibitory activity (IC_50_ = 0.64 μM) [28]. No synthetic approach has been reported to date.

#### 2.2.4. Aspericacids

**Figure 7:**
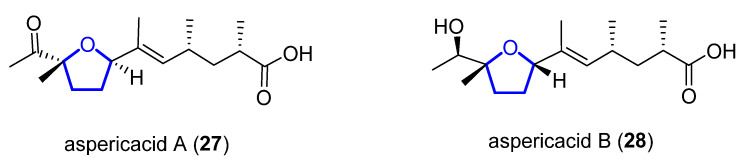
Structure of aspericacids A **27** and B **28**.

Aspericacids A (**27**) and B (**28**) were isolated in 2020 by Ding and He from the sponge-associated *Aspergillus* sp. LS78 [29]. Both compounds bear a 2,5-disubstituted tetrahydrofuran ring coupled with an unsaturated fatty acid (Figure 7). Their structure was determined by HRESIMS and 1D and 2D NMR spectroscopy, while their absolute configuration was established relying on electronic circular dichroism (ECD). Compound **27** presents a moderate inhibitory activity against *Candida albicans* and *Cryptococcus neoformans* with a MIC value of 50 μg/mL, although **28** has a weaker activity, MIC = 128 μg/mL. No synthetic approach has been reported to date.

## 3. Terpenes

### 3.1. Monoterpenes

#### 3.1.1. Pantofuranoids

**Figure 8:**
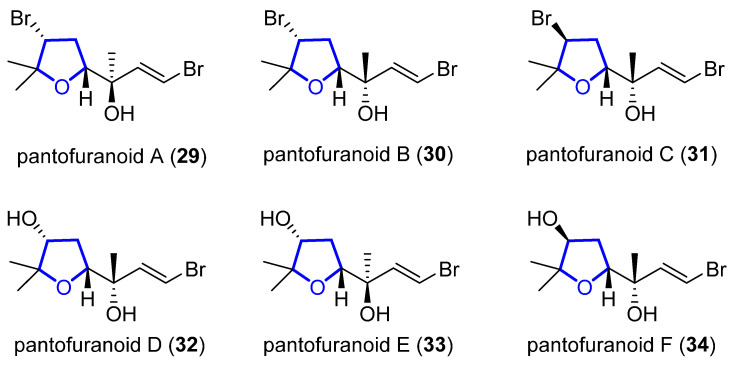
Proposed structure of pantofuranoids.

Pantofuranoids A–F (**29**–**34**) are monoterpenes that were isolated in 1996 from the Antarctic red alga *Pantoneura plocamioides* [30]. They are the first monoterpenes found to contain a tetrahydrofuran moiety, and their common framework (Figure 8) suggests that they all come from the same terpene precursor.

**Scheme 3:**
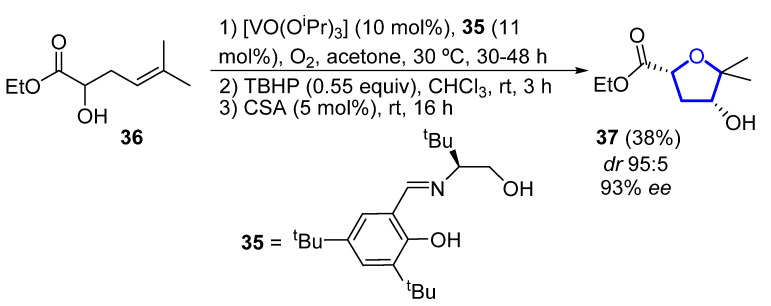
Vanadium-catalyzed synthesis of the tetrahydrofuranyl core of (−)-pantofuranoid E by Toste.

In 2006, Toste reported the enantioselective total synthesis of **(−)-33**, in which the key step is a vanadium-catalyzed sequential resolution/oxidative cyclization [31]. Using an in situ generated vanadium(V)–oxo complex with chiral tridentate Schiff base ligand **35** as catalyst, racemic homoallylic alcohol **36** was readily converted into 2,4-*cis*-substituted THF **37** (Scheme 3). The observed stereochemistry can be explained through a chair-like transition state in which coordination of the pseudo-equatorial ester group to the vanadium complex determines the selectivity of the *syn*-epoxidation step. Then, compound **37** was further elaborated to **(−)-33** in 6 steps and 29% overall yield from **37**.

#### 3.1.2. Furoplocamioids

**Figure 9:**
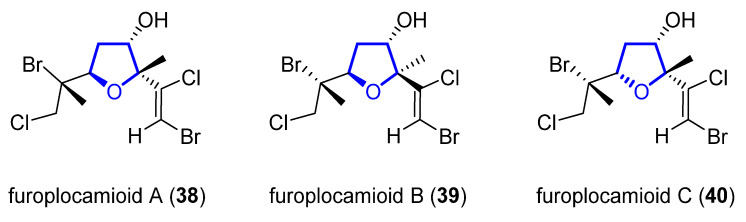
Structure of furoplocamioids A–C (**38–40**).

Furoplocamioids A–C **38–40** (Figure 9) are monoterpenes that were isolated in 2001 from the red marine alga *Plocamium cartilagineum* [32]. They bear an unusual polyhalogenated tetrahydrofuranyl ring. Their structural similarity to pantofuranoids suggests a close relationship between the species that produce them. This is an interesting fact, since *Plocamium cartilagineum* and *Pantoneura plocamioides* are classified in different orders, Gigartinales and Ceramiales. Therefore, a taxonomic revision could be required. Later, the Darias group determined the C7 relative stereochemistry by comparison with the NMR spectra of similar reported terpenes [33]. González-Coloma and coworkers found that **38** and **40** show antifeedant effects against *Leptinotarsa decemlineata*. It was shown that **40** was also an efficient aphid repellent (against *Mizuspersicae* and *Ropalosiphumpadi*) and selective insect cell toxicant. In addition, both compounds showed low mammalian toxicity and phytotoxic effects [34].

### 3.2. Sesquiterpenes

#### 3.2.1. Heronapyrrols

**Figure 10:**
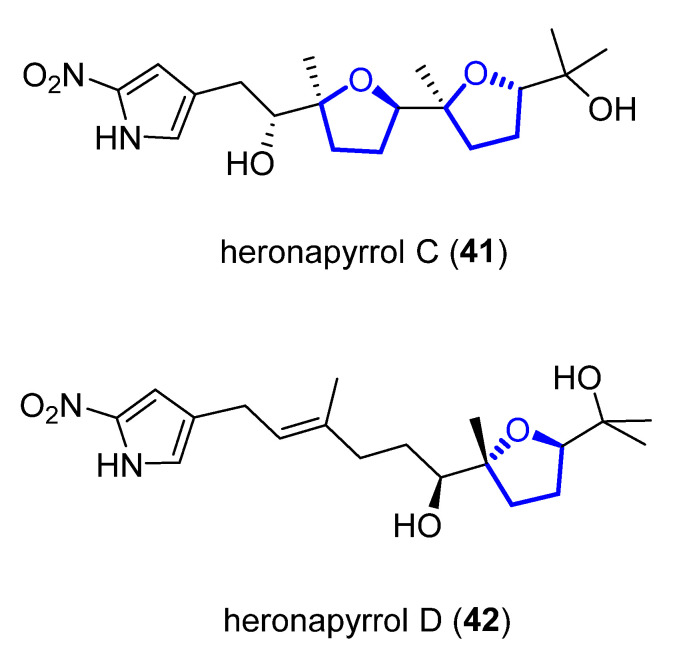
Structure of (+)-heronapyrrol C (**41**) and (+)-heronapyrrol D (**42**).

Heronapyrrols A–C are pyrroloterpenes that were isolated in 2010 from a marine *Streptomyces* sp. CMB-M0423 [35]. They present bioactivity against Gram-positive bacteria *Staphylococcus aureus* ATCC 9144 and *Bacillus subtilis* ATCC 6633 but no mammalian cytotoxicity. Heronapyrrol C (**41**), apart from the characteristic and unusual 2-nitropyrrol moiety of this family, presents a *bis*-tetrahydrofuran core. Later, Capon and Stark first synthesized and then isolated heronapyrrol D (**42**) (Figure 10) from the same marine-derived microbe [36]. Heronapyrrol D displays bioactivity against Gram-positive bacteria *Staphylococcus aureus* ATCC 25923 (IC_50_ = 1.8 μM), *Staphylococcus epidermidis* ATCC 12228 (IC_50_ = 0.9 μM), and *Bacillus subtilis* ATCC 6633 (IC_50_ = 1.8 μM). However, it is inactive against the Gram-negative bacteria *Pseudomonas aeruginosa* ATCC 10145 and *Escherichia coli* ATCC 25922. 

To determine the relative and absolute stereochemistry of **(+)-41**, Stark and coworkers proposed and synthesized the most likely stereostructure of its enantiomer **(−)-41**, based on a biomimetic polyepoxide cyclization [37]. The same authors also reported the preparation of a bioisosteric carboxylate analog of **(−)-41** [38]. 

The first total synthesis of **(+)-41** was reported in 2014 by Brimble and coworkers, who used as key steps to introduce the five stereogenic centers a Julia–Kocienski coupling, a Shi epoxidation, and a catalytic epoxide-opening reaction [39]. The same year, the first total synthesis of **(+)-42** was achieved by Capon and Stark using a similar approach [36]. In 2016, Brimble and Furkert reviewed the isolation and synthesis of this family of compounds [40].

**Scheme 4:**
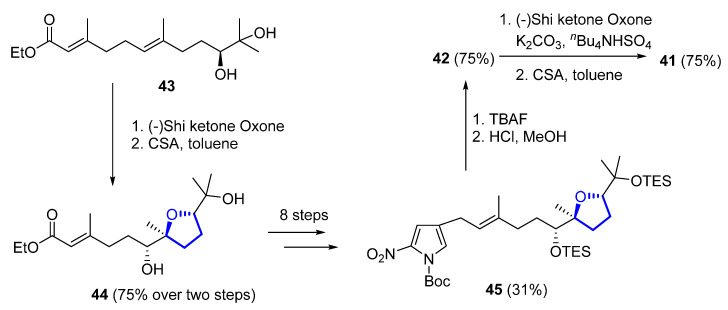
Synthesis of (+)-heronapyrrol C (**41**) and (+)-heronapyrrol D (**42**).

Later on, the same authors reported another total synthesis for both **(+)-41** and **(+)-42 [41]**. Shi epoxidation of diol **43**, followed by CSA-catalyzed epoxide opening and cyclization, produces diastereomerically pure **44** in 75% yield over two steps. A further eight steps, with 31% yield over them, produces intermediate **45**, which deprotection gives (+)-heronapyrrol D (**42**). Epoxidation of **42** with a Shi ketone catalyst, followed by CSA-catalyzed epoxide opening, produced enantiomerically pure (+)-heronapyrrol C (**41**) in 75% yield (Scheme 4).

Other synthetic approaches towards the 2-nitropyrrole system have been investigated by Brimble and Furkert, finding that Sonogashira coupling of 4-iodo-2-nitropyrrole with the appropriate alkyne was more effective than an approach relying on Stille coupling [42].

#### 3.2.2. Kumausallene and Kumausyne

**Figure 11:**
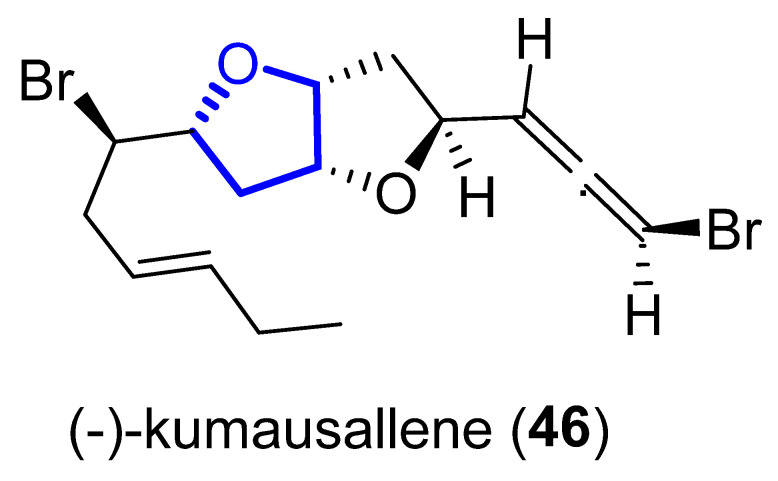
Structure of kumausallene.

(−)-Kumausallene (**46**) was isolated in 1983 from the marine red alga *Laurencia nipponica* Yamada [43]. This compound belongs to a family of non-isoprenoid sesquiterpenes that contain a 2,6-dioxa-bicyclo [3.3.0]octane core with an *exo*-cyclic bromoallene (Figure 11). 

**Scheme 5:**
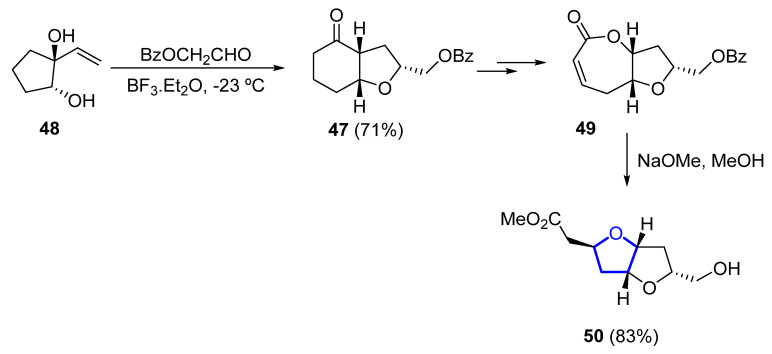
Synthesis of the dioxabicyclo [3.3.0]octane unit of (±)-kumausallene (**46**) by Overman.

In 1993, the first total synthesis of **(±)-46** was reported by Overman, who chose a hexahydrobenzofuranone **47** (obtained by a Prins cyclization–pinacol rearrangement from **48**) as the key intermediate for the construction of the *bis*-tetrahydrofuran unit (Scheme 5). Further transformation of **47** into bicyclic lactone **49** (within three further steps) and final methanolisis and tandem cyclization of the corresponding hydroxyester provided, in good yield, the desired dioxabicyclo [3.3.0]octane **50** [44].

**Scheme 6:**
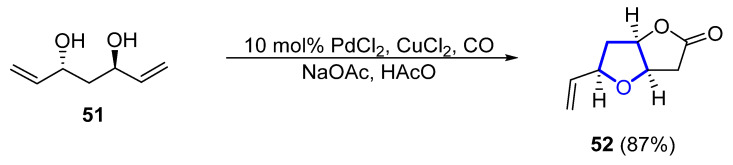
Synthesis of the tetrahydrofuranyl ring of **(−)-46** through desymmetrization by Tang.

**Figure 12:**
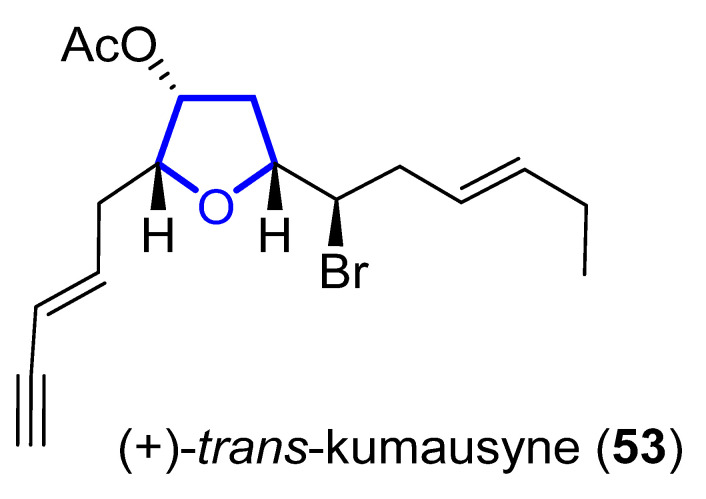
Structure of (+)-*trans*-Kumausyne (**53**).

In 2011, a synthetic approach for **(−)-46** by Tang employed a desymmetrization strategy for the formation of the 2,5-*cis*-substituted THF ring [45]. *C_2_*-symmetric diol **51** is desymmetrized by a palladium-catalyzed cascade reaction to form lactone **52** in 87% yield (Scheme 6). The total synthesis comprised just 12 steps from commercial acetylacetone. In 2015, Ramana et al. published a different formal total synthesis of **(−)-46** based on a chiral pool approach [46].

(+)-*Trans*-Kumausyne (**53**) (Figure 12) is a halogenated non-isoprenoid sesquiterpene isolated in 1983 from red alga *Laurencia nipponica* Yamada [47]. Its first total synthesis was achieved in 1991 by Overman and coworkers [48]. A review covering the synthetic approaches towards kumausallene and kumausyne, and other natural products containing a 2,3,5-trisubstituted tetrahydrofuran moiety, was published by Fernandes in 2020 [18].

### 3.3. Diterpenes

#### 3.3.1. Darwinolide

**Figure 13:**
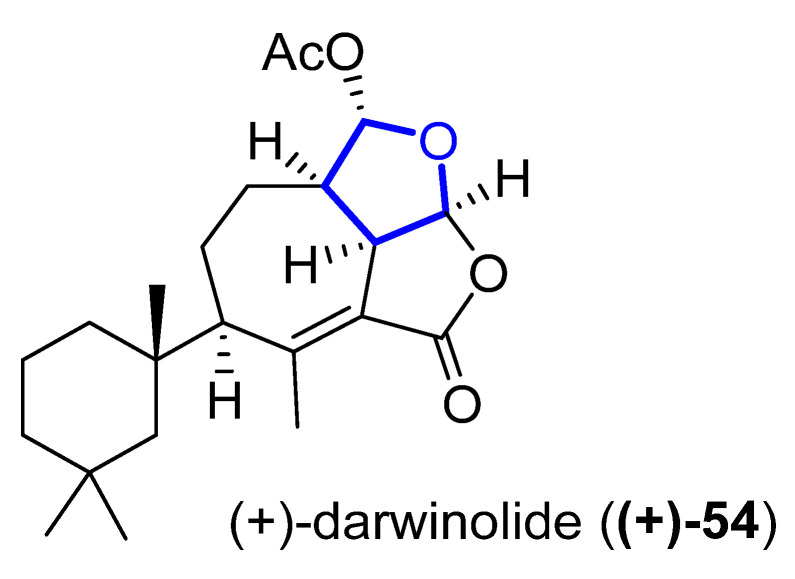
Structure of (+)-darwinolide (**(+)-54**).

(+)-Darwinolide (**54**) (Figure 13) is a diterpene isolated in 2016 by Baker from the Antarctic Dendroceratid sponge *Dendrilla membranosa* [49]. It presents fourfold selectivity against a biofilm phase of methicillin-resistant *Staphylococcus aureus* (IC_50_ of 33.2μM), compared to the planktonic phase (with the higher MIC of 132.9 μM). This interesting property and its low mammalian cytotoxicity (IC_50_ of 73.4 mM) against a J774 macrophage cell line turn darwinolide into a possible scaffold for antibiofilm-specific antibiotics. Additionally, it was found to have modest activity (11.2 μM) against *L. donovani*-infected macrophages [50].

**Scheme 7:**
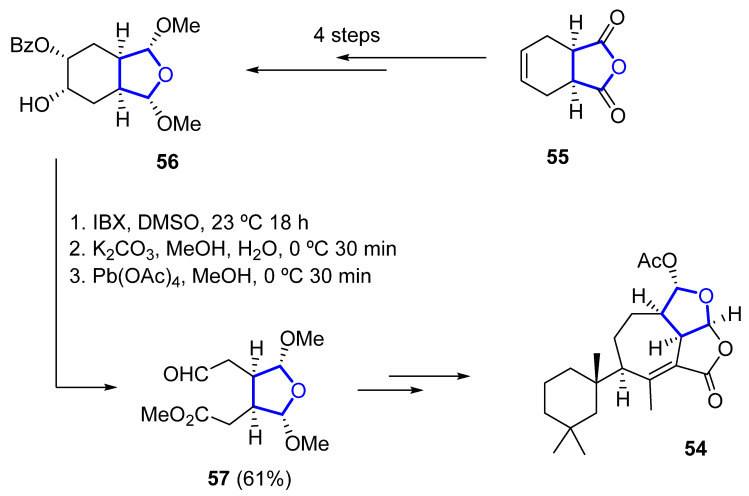
Synthesis of the key THF-containing intermediate in Christmann’s total synthesis of darwinolide.

Its total synthesis was reported in 2019 by Christmann [51]. The required tetrahydrofuranyl ring is installed starting from the commercially available anhydride **55**, which is converted to the 2,5-dimethoxylated tetrahydrofuran **56** in four steps. A sequence of oxidation (with o-iodoxybenzoic acid), saponification, and Criegee oxidation with Pb(OAc)_4_ is then used to convert **56** into **57** in 61% yield over three steps (Scheme 7). A further 14 linear steps are needed to complete the total synthesis of **54**, with an overall yield of 1.4%.

#### 3.3.2. Uprolides

**Figure 14:**
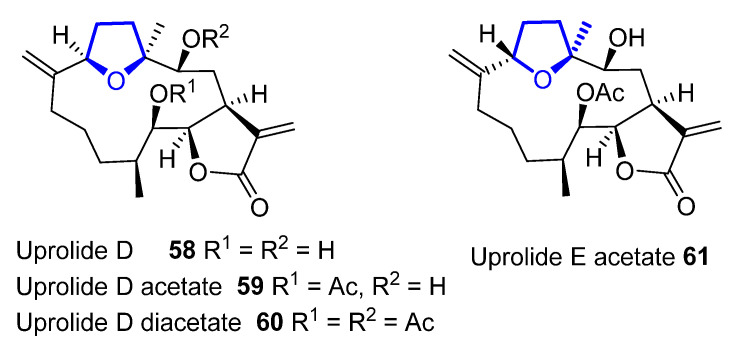
Structure of tetrahydrofuran-containing members of the uprolide family.

Cembranolides are a family of compounds related to cembrene, which is a 14-membered macrocyclic diterpene with multiple (*E*)-double bonds. Among them, uprolides are a subfamily of compounds named after the University of Puerto Rico. Uprolides A–G were isolated in 1995 by Rodríguez and coworkers from the Caribbean gorgonian *Eunicea Mammosa* and their structure was assigned by spectroscopic methods and chemical interconversion [52,53]. They are the first natural cembranolides from a Caribbean gorgonian that bear a double bond at C6 or C8. While uprolides A–C show an epoxy moiety, uprolides D–G seemed to contain a tetrahydrofuran moiety instead. Uprolides D (**58**), D acetate (**59**), D diacetate (**60**), and E acetate (**61**) (Figure 14) present a moderate cytotoxicity against HeLa cells ((IC_50_ = 2.5 to 5.1 µg/ ml). Moreover, **59** shows cytotoxicity against the following human tumor cell lines: CCRF-CEM T-cell leukemia (IC_50_ = 7.0 μg/mL), HCT-116 colon cancer (IC_50_ = 7.0 μg/mL), and MCF-7 breast adenocarcinoma (IC_50_ = 0.6 μg/mL).

Later structural revisions determined the presence of a tetrahydropyran ring, instead of the previously proposed tetrahydrofuran, in uprolides F diacetate and G acetate, hypotheses that were confirmed by asymmetric total synthesis of these natural products [54,55,56,57].

**Scheme 8:**
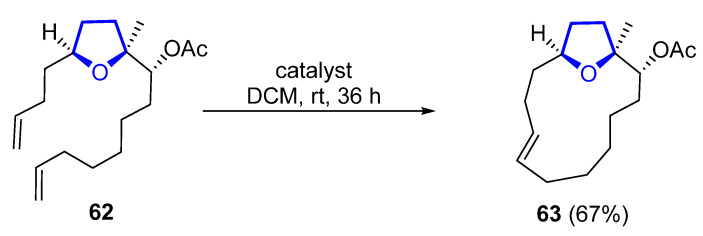
Synthesis of macrocyclic core of uprolide D by Ramana.

In 2007, a synthetic approach to obtain the macrocyclic core of **58** was proposed by Ramana [58]. The formation of the macrocyclic core is produced by ring-closing metathesis (RCAM) using a Grubbs’ first-generation catalyst. The RCAM of acetate **62** produces 13-membered macrocyclic **63** in 67% yield (Scheme 8). The macrocyclic core of uprolide E could not be synthesized using the same methodology.

Marshall developed a synthetic route for C1/C14 *bis*-epimer of **58** in which the macrocyclization is produced by an intramolecuar Barbier reaction [59]. Some years later, other members of the uprolide family, which lack the tetrahydrofuran ring, have been isolated from the gorgonian octocoral *Eunicea succinea* [60].

### 3.4. Triterpenes

#### 3.4.1. Intricatetraol

**Figure 15:**
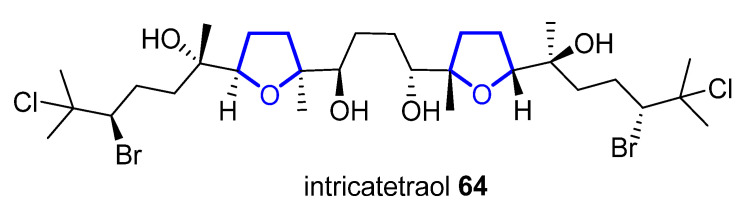
Structure of (+)-intricatetraol **64**.

Intricatetraol (**64**) is a halogenated triterpenoid with a *C_2_* symmetry that was isolated in 1993 from the red alga *Laurencia intricata* (Figure 15). Suzuki and coworkers observed that a crude fraction of the extract showed cytotoxic activity against P388 leukemia cells with an IC_50_ value of 12.5 μg/mL. Nevertheless, pure intricatetraol was no longer active after chromatography. At first, its stereostructure was proposed as being based on its hypothetical biogenesis [61]. In 2006, Morimoto confirmed this assignment by synthesizing a degradation product of intricatetraol through a two-directional synthesis [62].

**Scheme 9:**
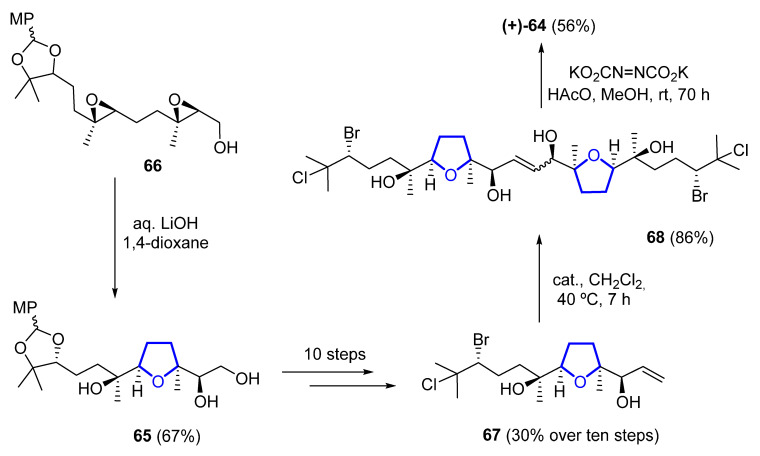
Synthesis of (+)-intricatetraol (**(+)-64**).

One year later, Morimoto completed the asymmetric total synthesis of **(+)-64** and, thus, determined its absolute configuration [63]. The tetrahydrofuran ring **65** was stereospecifically constructed by treatment of diepoxyalcohol **66** with lithium hydroxide aqueous solution. A further 10 steps (with an accumulated yield of 30%) were needed to obtain intermediate **67**, which was then dimerized to afford **68** by olefin metathesis with a second-generation Grubbs catalyst. Final diimide reduction of intermediate **68** produced **(+)-64** (Scheme 9).

#### 3.4.2. Omaezakianol

**Figure 16:**
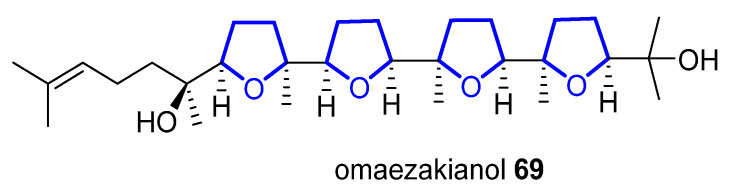
Structure of (+)-omaezakianol (**69**).

Omaezakianol (**69**) (Figure 16) is a squalene-derived triterpene polyether that was isolated in 2008 from the red alga *Laurencia omaezakiana* by Morimoto and coworkers [64]. The same group rapidly reported its first asymmetric total synthesis and, therefore, stablished its absolute configuration [65]. Key steps were olefin cross-metathesis and an epoxide-opening cascade.

**Scheme 10:**
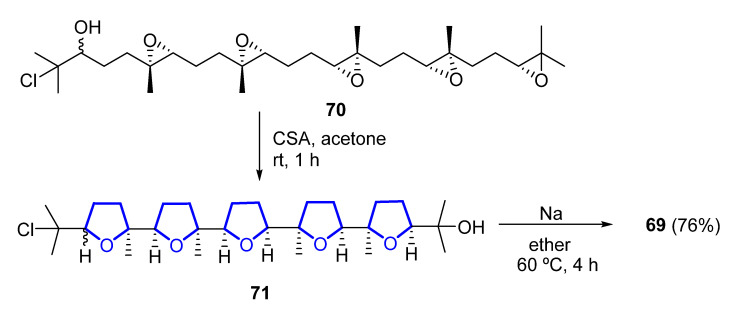
Synthesis of (+)-omaezakianol **69** by Corey via an epoxide-initiated cationic cascade.

In 2010, Corey reported a short total synthesis via a biomimetic epoxide-initiated cationic cascade reaction [66]. Compound **70** was treated with camphorsulfonic acid (CSA) to induce the epoxide-opening cascade cyclization, producing **71**. Reduction of **71,** with sodium in acetone under reflux, formed the terminal double bond with opening of the THF ring, affording **69** in 76% yield (Scheme 10). Thus, the synthesis was accomplished in just six steps from squalene.

**Scheme 11:**
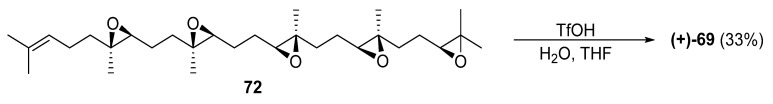
Synthesis of (+)-omaezakianol (**(+)-69**) by a biomimetic epoxide-opening cascade.

Another biomimetic epoxide-opening cascade for the synthesis of **(+)-69** was reported in 2013 by Morimoto and coworkers [67]. The cascade, which mimics the direct hydrolysis mechanism of epoxide hydrolases, begins with 5-*exo* cyclization of the terminal epoxide triggered by Brønsted acid catalysis. Intermediate **72** then undergoes an epoxide-opening cascade reaction with TfOH to afford **(+)-69** in 33% yield (Scheme 11).

#### 3.4.3. Longilenes

Longilenes are triterpene polyethers that were first isolated from the wood of *Eurycoma longifolia* in the form of (−)-longilene peroxide (**(−)-73**) [68,69]. In 2001, Morimoto accomplished its total synthesis [70] and determined its absolute configuration. The same author also developed a biomimetic synthesis of the C9–C16 fragment of oxasqualenoids in an enantioselective manner [71]. This chiral building block could be used as an advanced intermediate for the synthesis of different polyethers, such as teurilene, longilene peroxide, or glabrescol.

**Figure 17:**
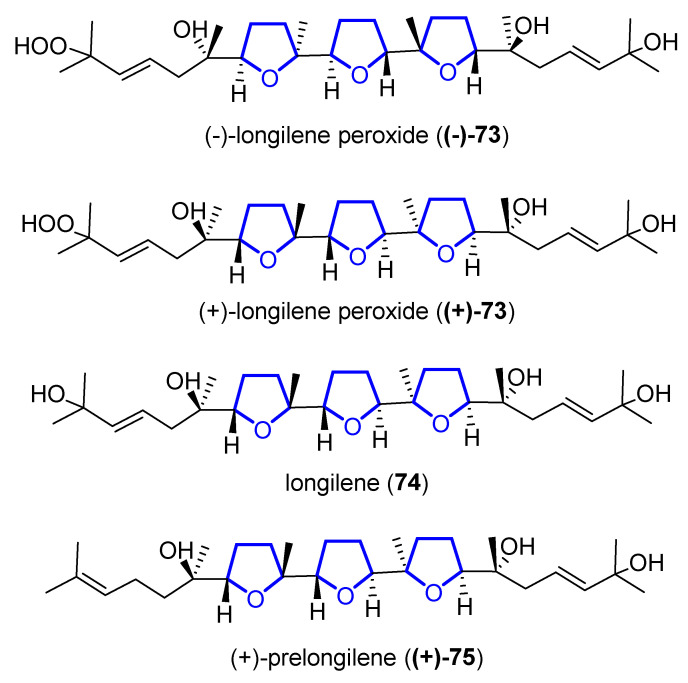
Structure of (−)-longilene peroxide (**(−)-73**), (+)-longilene peroxide (**(+)-73**), longilene (**74**), and the derivative (+)-prelongilene (**(+)-75**).

In 2018, Fernández and Daranas reported the isolation of other members of the family, namely (+)-longilene peroxide (**(+)-73**), longilene (**74**), and the derivative (+)-prelongilene (**(+)-75**), from the red seaweed *Laurencia viridis* [72]. These compounds present Ser/Thr protein phosphatase 2A inhibitory activity (Figure 17). To date, no synthetic approaches have been reported.

#### 3.4.4. Laurokanols and Yucatecone

**Figure 18:**
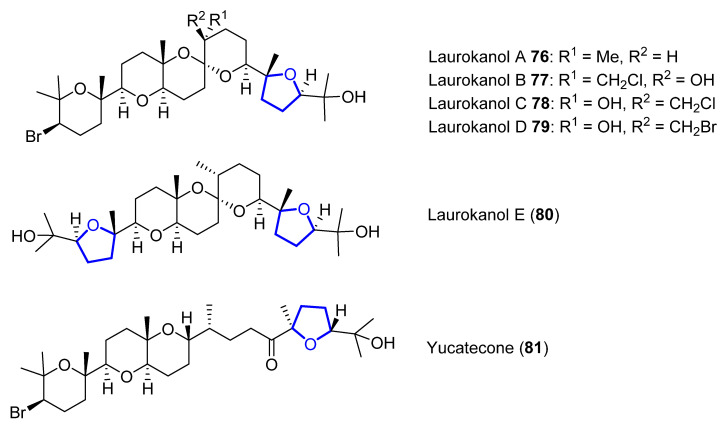
Structures of laurokanols A–E (**76**–**80**) and yucatecone (**81**).

In 2021, laurokanols A–E (**76–80**) and yucatecone (**81**) (Figure 18), polyether triterpenes, were isolated from the red alga *Laurencia viridis* [73]. Laurokanols have an unprecedented tricyclic core with an [6,6]-spiroketal system. Yucatecone is the first compound of this series with an *R* configuration at the C14 position. A biogenetic model, supported by DFT calculations, was then postulated for the biogenesis of yucatecone.

#### 3.4.5. Thyrsenol

**Figure 19:**
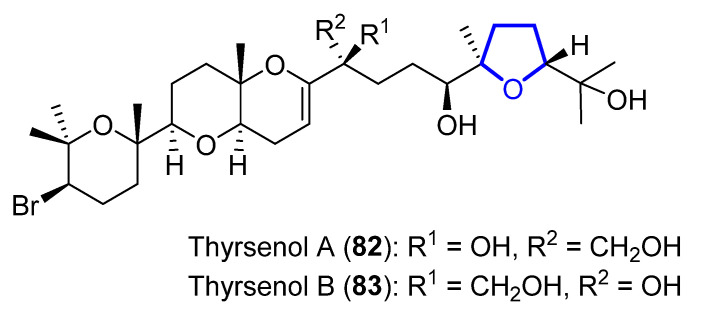
Structure of thyrsenol A (**82**) and B (**83**).

Thyrsenol A (**82**) and B (**83**) (Figure 19) are polyether squalene derivatives that were isolated by Norte and coworkers in 1997 from the red alga *Laurencia viridis* [74]. Although both compounds show high activity against murine lymphoid neoplasm P-388 cells, compound **83** induced significantly higher inhibitory effects [75]. Other related compounds, such as thyrsiferol derivatives and dehydrovenustatriol, were found to be even more active. Therefore, it was postulated that the presence of a flexible chain around C14 to C19, and its configuration, are of particular importance for the bioactivity of these compounds. Later, Fernández and coworkers also found **83** to have protein phosphatase PP2A inhibitory activity [76]. The activity was comparable to that of dehydrothyrsiferol and thysiferyl-23-acetate, concluding that a hydroxyl group at C15 or C16 is a key factor for their intrinsic activity. In 2011, the isolation of other derivatives has been reported [77].

#### 3.4.6. Saiyacenol

**Figure 20:**
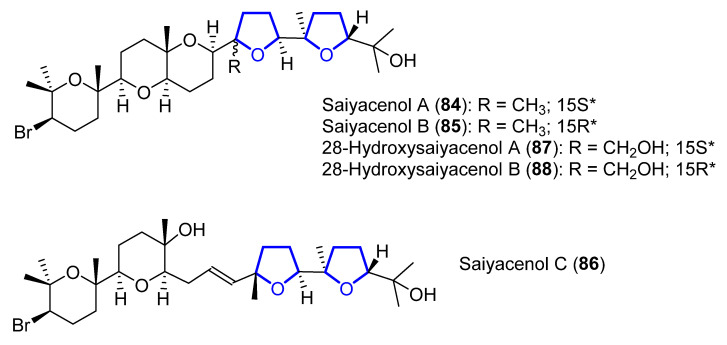
Structure of saiyacenols A–C (**84**–**86**) and their hydroxylated derivatives **87** and **88**.

Saiyacenols A (**84**) and B (**85**) (Figure 20) are triterpene polyethers that were isolated in 2012 from the red alga *Laurencia viridis*. Both inhibit cell proliferation of various human tumor cell lines (MM144 (human multiple myeloma), HeLa (human cervical carcinoma), CADO-ES1 (human Ewing’s sarcoma), and show the best inhibitory activity against Jurkat (human T-cell acute leukemia) [78]. In 2015, saiyacenol C (**86**), and two hydroxylated derivatives **87** and **88**, were isolated from the same alga [79]. Although saiyacenols showed no activity toward a range of bacteria and fungal strains, compounds **85** and **86** avoid *Navicula* cf. *salinicola* and *Cylindrotheca* sp. growth, while compounds **87** and **88** were also active against germination of *Gayralia oxysperma*.

More recently, Piñero and Fernández evaluated a range of natural and semisynthetic terpenoid polyethers against protozoan parasites of the *Trypanosoma* and *Leishmania* genera [80]. Both **84** and **85** have anti-protozoal activity against *Leishmania amazonensis* (IC_50_ = 12.96 and 10.32 μM, respectively). Interestingly, saiyacenol A **84** was also effective against the highly resistant *Trypanosoma cruzi* (IC_50_ = 13.75 ± 2.28 μM). The semisynthetic 28-iodosaiyacenol B showed a high value (IC_50_ = 5.40 ± 0.13 μM) against *Leishmania amazonensis* and no toxicity to murine macrophage J774.A1. This turns it into a possible scaffold for antikinetoplastid drugs, as the data are comparable to those of the reference drug miltefosine (IC_50_ = 6.48).

**Scheme 12:**
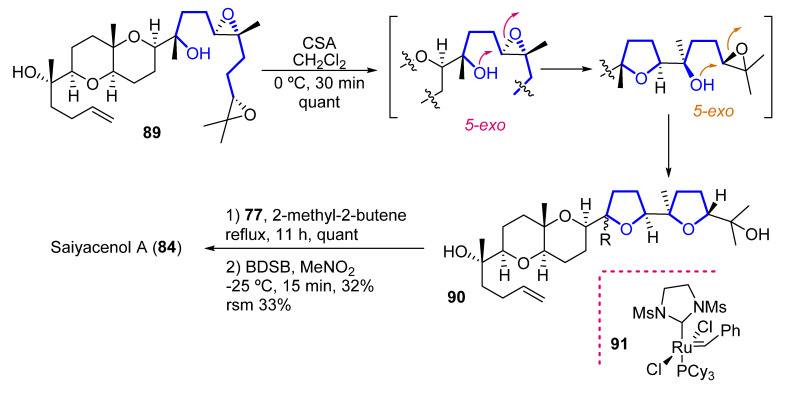
Synthesis of saiyacenol A by Nishikawa and Morimoto.

Very recently, Nishikawa and Morimoto reported the asymmetric total synthesis of saiyacenol A, along with that of the related Aplysiol B [81]. In their research, an epoxide-opening cascade was used to form both THF rings in the same step (Scheme 12). Thus, from advanced precursor **89**, treatment with CSA provoked two sequential 5-*exo* openings to form the oxygenated rings in *bis*-tetrahydrofuran **90**. The last step consisted of cross-methathesis with the ruthenium catalyst **91** and later bromoetherification with BDSB. Preliminary cytotoxicity was tested against P388, HT-29, and HeLa tumor cell lines, showing values of 5.4, 85, and >100 μM, respectively.

#### 3.4.7. Teurilene

**Figure 21:**
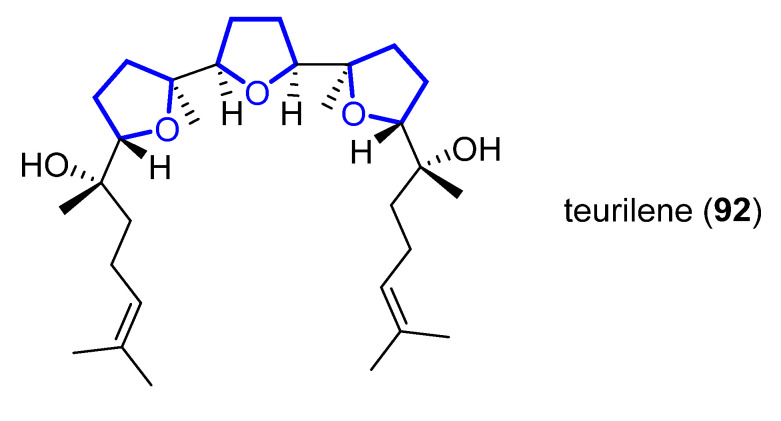
Structure of teurilene **92**.

Teurilene (**92**) (Figure 21) is a triterpene polyether that was isolated in 1985 from the red alga *Laurencia obtusa* by Kurosawa and coworkers [82]. It has three linked 2,5-disubstituted THF rings and, even though it has eight stereogenic centres, it is an achiral compound due to its *meso*-symmetry (*C_s_*). Compound **92** has a remarkable cytotoxic activity against KB cells (IC_50_ = 7.0 μg mL^−1^) [69]. Synthetic approaches and routes up to 2014 are commented on in a previous review [83].

#### 3.4.8. Thyrsiferol

**Figure 22:**
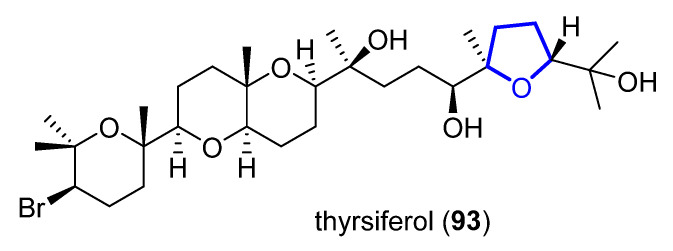
Structure of thyrsiferol (**93)**.

Thyrsiferol (**93**) is a polyoxygenated triterpenoid ether that was isolated in 1978 from the red alga *Laurencia thyrsifera* [84]. Its absolute stereochemistry (Figure 22) was determined in 1986 when venustatriol was isolated, since the latter could be crystallized and characterized by X-ray diffraction [85]. During the next two decades, a plethora of thyrsiferol derivatives have been isolated. Their biological profiles (cytotoxic, anti-viral, anti-tumor) have attracted much attention and several syntheses were published and reviewed in 2008 [86].

Thyrsiferol has later been found to inhibit hypoxia-induced hypoxia-inducible factor-1 (HIF-1) activation in T47D human breast tumor cells, as well as to inhibit a mitochondrial ETC complex I and show tumor cell line-selective time-dependent inhibition of cell viability/proliferation [77]. In addition, Piñero and Fernández recently reported it to be active against *Acanthamoeba castellanii* trophozoites (IC_50_ = 13.97 μM). Its derivative, 22-hydroxydehydrotyrsiferol, was similarly active (IC_50_ = 17.00 μM) and both were not toxic against murine macrophages, which makes them potential leads for the discovery of novel amoebicidal substances [87].

#### 3.4.9. Rhabdastins

**Figure 23:**
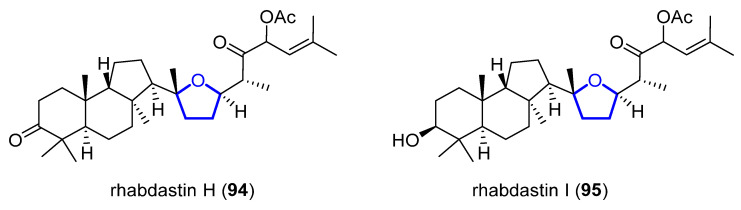
Structure of rhabdastins H (**94**) and I (**95**).

The first members of this family, Rhabdastins A-G, were first isolated in 2010 by Iwagawa from the sponge *Rhabdastrella globostellata* [88]. They belong to the group ofisomalabaricane triterpenes. In 2021, the tetrahydrofuran-containing rhabdastins H (**94**) and I (**95**) (Figure 23) were isolated from the sponge *Rhabdastrella* sp. [89]. They are the first marine isomalabaricanes that present a tetrahydrofuran unit in their structure. Both compounds show antiproliferative effect against K562 (IC_50_ 11.7 and 9.8 μM, respectively) and Molt4 (IC_50_ 16.5 and 11.0 μM) leukemic cells.

### 3.5. Meroterpenes

#### 3.5.1. Alisiaquinones and Alisiaquinol

**Figure 24:**
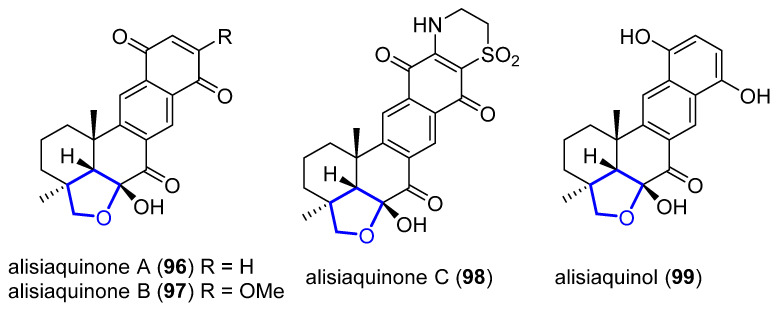
Structure of alisiaquinones A–C (**96–98**) and alisiaquinol (**99**).

Alisiaquinones A–C (**96**–**98**) and alisiaquinol (**99**) (Figure 24) are meroterpenes that were isolated in 2008 from a New Caledonian deep-water sponge [90]. They display mild antimalarial activity, but the high level of toxicity (100% and 80% mortality, respectively) shown in in vivo assays limited their interest as antimalarial agents. Nevertheless, their structure can be an inspiration for the development of related structures towards novel antimalarial drugs.

#### 3.5.2. Tricycloalterfurenes

**Figure 25:**
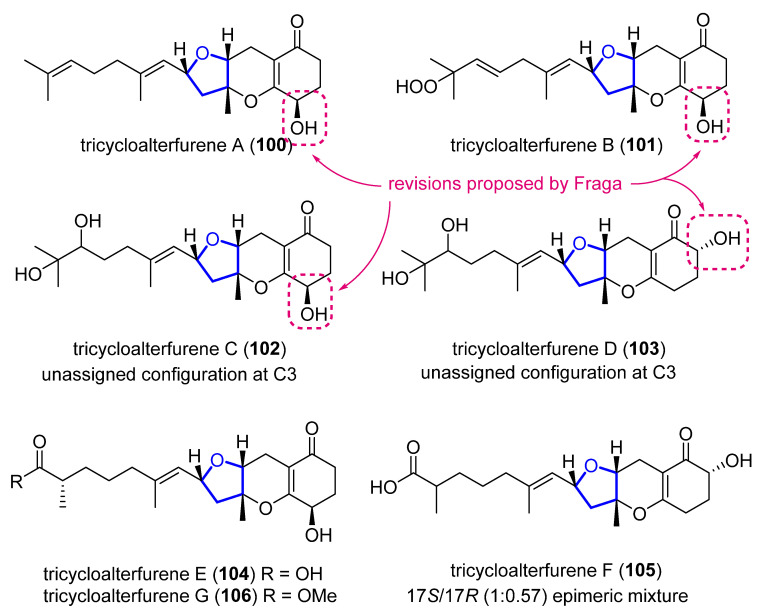
Structure of tricycloalterfurenes and proposed structural revisions (in pink) by Fraga.

Tricycloalterfurenes A–D (**100**–**103**) were isolated in 2017 from the culture extract of an *Alternaria alternata* strain (k21-1) isolated from the surface of the marine red alga *Lomentaria hakodatensis* [91]. These meroterpenes present activity against three phytoplankton (*Chattonella marina*, *Heterosigma akashiwo*, and *Prorocentrum donghaiense*) and one marine zooplankton (*Artemia salina*). The higher activity of tricycloalterfurene A (64, 37, 46%, respectively, against the phytoplankton species) indicates that hydroxylation at C2 or C3 negatively influences the activity against these organisms. Later, Oh and Shin reported the isolation, from a marine-derived *Stemphylium* sp. fungus, of tricycloalterfurenes E–G (**104**–**106**) [92]. Very recently, Fraga proposed some structural revisions [93]. Regarding tricycloalterfurenes A–C, the correct configuration of the hydroxyl group would be *4R* (Figure 25), comparing the NMR data with that of guignardone T [94]. With respect to trycicloalterfurene D, the correct configuration was proposed to be 6-β-OH (*6R*), also by NMR data comparison to similar systems [95]. To date, no synthetic approaches have been reported.

#### 3.5.3. Marinoterpins

**Figure 26:**
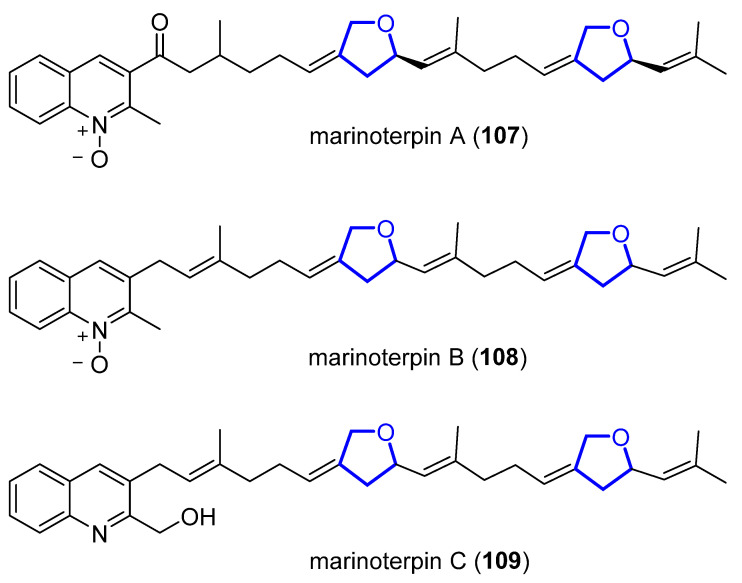
Structure of marinoterpins A–C (**107**–**109**).

Marinoterpins A–C (**107**–**109**) (Figure 26) are linear merosesterterpenoids that were recently isolated (2021) by Winter and Fenical from the marine-derived actinomycete bacteria *Streptomyces* sp. AJS-327 and CNQ-253 [96]. Due to their similarity to the aurachin family of compounds, a marinoterpin biosynthetic cluster (*mrt*) was identified. Thus, a biosynthetic route for the 3-geranylfarnesyl-2-methylquinoline core was proposed, although the reactions that lead to the tetrahydrofuran rings as well as the N-oxidation still remain unknown.

## 4. Other Tetrahydrofuran Derivatives

### 4.1. Substituted Tetrahydrofurans

#### 4.1.1. Pachastrissamine (Jaspine B)

**Figure 27:**
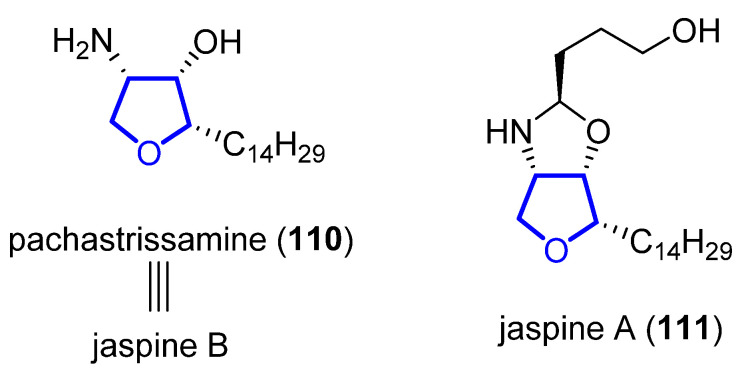
Structures of pachastrissamine (jaspine B, **110**) and jaspine A (**111**).

Pachastrissamine (**110**) was first isolated by Higa and coworkers from a marine sponge of the genus *Pachastrissa* [97]. A year later, Debitus reported its isolation from another sponge *Jaspis* sp. and, thus, named the compound jaspine B [98]. Other related compounds were also isolated, including jaspine A (**111**) (Figure 27). Pachastrissamine is a sphingolipid—in particular, a derivative from anhydrophytosphingosine. Its biological activity and synthetic endeavors until 2016 were summarized by Martinková in a mini-review [99].

#### 4.1.2. Myrothecols

**Figure 28:**
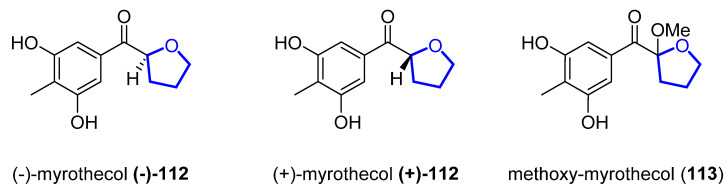
Structure of (−)-1*S*-myrothecol (**(−)-112**), (+)-1*R*-myrothecol (**(+)-112**), and methoxy-myrothecol (**113**).

In 2020, (−)-1*S*-myrothecol (**(−)-112**), (+)-1*R*-myrothecol (**(+)-112**), and methoxy-myrothecol (**113**) (Figure 28) were isolated from deep-sea fungus *Myrothecium sp.* BZO-L062 [100]. Enantiomers **(−)-112** and **(+)-112** were separated by normal-phase chiral HPLC, and their absolute configurations were established by ECD spectra. Compounds **(−)-112** and **(+)-112** display anti-inflammatory activity, inhibit nitric oxide formation in lipopolysaccharide-treated cells (RAW264.7), and present antioxidant activity in the 2,2-azino-*bis*(3-ethylbenzothiazoline-6-sulfonic acid) and oxygen-radical absorbance capacity assays.

#### 4.1.3. Astronypyrone, Astronyquinone, and Astronyurea

**Figure 29:**
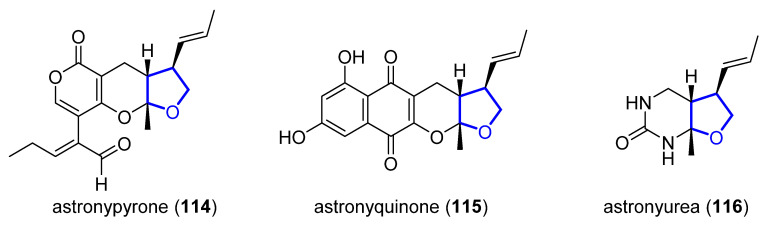
Structure of astronypyrone (**114**), astronyquinone (**115**), and astronyurea (**116**).

In 2016, astronypyrone (**114**), astronyquinone (**115**), and astronyurea (**116**) (Figure 29) were isolated from the marine fungus *Astrosphaeriella nypae* BCC 5335 [101]. Compound **115** shows weak antituberculosis activity (with a MIC value of 50 μg/mL) and presents cytotoxicity against African green monkey kidney fibroblast cell lines (IC_50_ = 17.4 μg/mL).

#### 4.1.4. Sinularones

**Figure 30:**
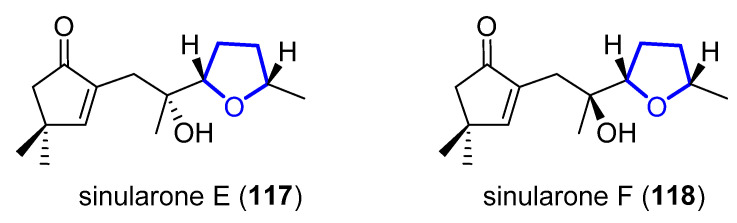
Structure of sinularones E (**117**) and F (**118**).

Sinularones A-I were isolated in 2012 from the marine soft coral *Sinularia* sp. [102]. Their structures were elucidated by IR, MS, CD, 1D, and 2D NMR. Among them, sinularones E (**117**) and F (**118**) contain a tetrahydrofuran moiety (Figure 30).

**Scheme 13:**
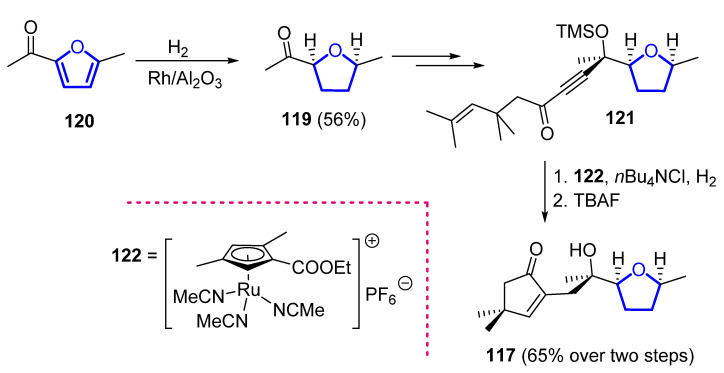
Synthesis of sinularone E (**117**). A similar strategy was applied to sinularone F (**118**).

Their first total synthesis, based on a hydrogenative metathesis of enynes, was reported in 2020 by Fürstner and coworkers [103]. The required 2,5-*cis*-tetrahydrofuran derivative **119** was readily obtained by hydrogenation (over Rh/Al_2_O_3_) of commercial furane **120**. Three further steps were needed to obtain silyl ether **121**, which in the presence of ruthenium catalyst **122** and H_2_ undergoes a hydrogenative metathesis. Later deprotection with TBAF produces **117** with 65% yield over both steps. Sinularone F (**118**) synthesis utilizes the same strategy (Scheme 13).

#### 4.1.5. (+)-Varitriol

**Figure 31:**
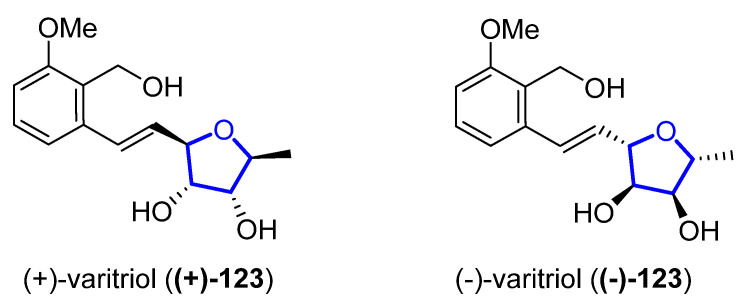
Structure of (+)-varitriol (**(+)-123**) and (−)-varitriol (**(−)-123**).

(+)-Varitriol (**(+)-123)** (Figure 31) was isolated in 2002 from a marine-derived strain of the fungus *Emericella variecolor* [104]. Its structure and relative stereochemistry were determined by NMR studies. It displays low cytotoxic activity against leukemia, ovarian, and colon cells, but its response to renal, CNS, and breast cancer cell lines was very promising (of the range of GI_50_ = 1.63–2.44·10^−7^ M). In 2006, Jennings and coworkers achieved the first total synthesis of its enantiomer **(-)-123** and, thus, determined its absolute stereochemistry [105].

**Scheme 14:**
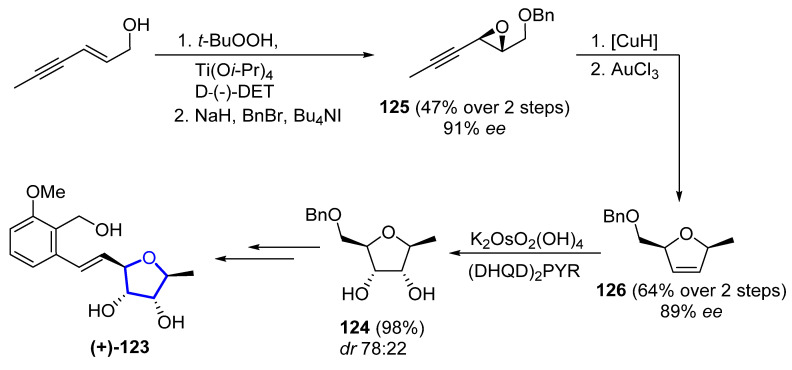
Modular synthesis of (+)-varitriol (**(+)-123**) by Krause.

Its activity towards the mentioned cancer cell lines attracted the attention of synthetic chemists; different synthetic approaches for **(+)-123** appeared in the next few years [106]. Taylor employed a known tetrahydrofuran-2,3,4-triol derivative as the starting material for the synthesis (in three further steps) of the desired **(−)-123** and its 3′-epi-derivative [107]. Srihari also achieved the synthesis of **(−)-123**, **(+)-123**, and its 6′-epi-derivative starting from commercial D-(−)-ribose [108,109]. Krause performed a modular synthesis of **(+)-123** in 10 steps, with an overall yield of 6,4% [110]. The key tetrahydrofuran with the appropriate four stereocenters **124** was prepared in four linear steps from a known enyne. Thus, Sharpless epoxidation and benzylation of (*E*)-hex-2-en-4-yn-1-ol provided propargyl epoxide **125** in 47% yield over two steps. A copper hydride-catalyzed reduction of **125**, followed by a gold-catalyzed cycloisomerization, furnished the key dihydrofuranyl intermediate **126**, with two of the required stereogenic centers. Final Sharpless dihydroxylation of **126** afforded **124** as a satisfactory 78:22 mixture of diastereoisomers. The desired natural product was obtained in a further four steps (Scheme 14).

**Scheme 15:**
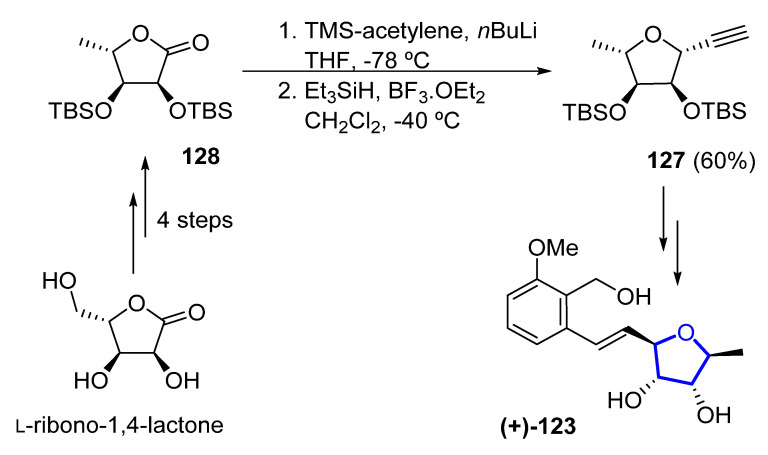
Protecting group-directed nucleophilic addition for the synthesis of (+)-varitriol (**123**).

A recent example of the total synthesis of **(+)-123** was reported by Cordero-Vargas [111]. In this approach, the tetrahydrofuranyl key intermediate **127**, bearing the four precise sterocenters, was obtained from commercial L-ribono-1,4-lactone in six steps. The key step in this process is the stereocontrolled nucleophilic addition to five-membered oxocarbenium ions directed by the protecting groups. Thus, TBS-protected lactone **128**, obtained in four conventional steps from L-ribono-1,4-lactone, undergoes acetylide addition; a subsequent Lewis acid promoted oxocarbenium ion formation. The following stereoselective hydride attack provides tetrahydrofuran **127** as a single diastereomer in 60% yield. The final synthesis of **(+)-123** is achieved in a further four steps with 31% yield over them (Scheme 15).

**Scheme 16:**
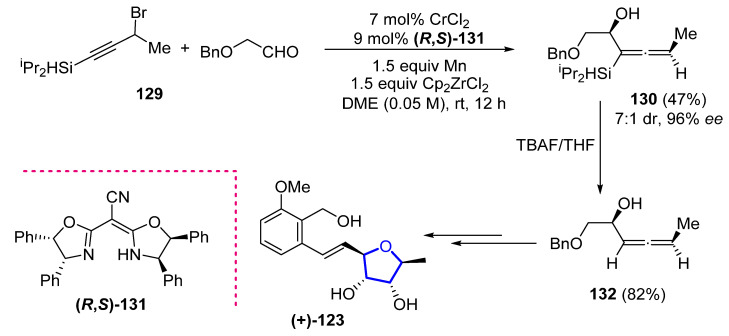
Application of allenylation of aldehydes to the formal synthesis of (+)-varitriol by Wang.

Later on, Qin developed a direct cross-coupling between terminal alkynes and glycosyl acetates, which was applied to the formal synthesis of **(+)-123** and analogues [112]. More recently, Wang and coworkers reported another formal synthesis of **(+)-123** [113]. They developed a chromium-catalyzed enantioconvergent allenylation of aldehydes to synthesize α-allenols from racemic propargyl halides. Starting from silylated propargyl bromide **129** and benzyloxyacetaldehyde, allenol **130** can be accessed through the developed procedure. The chromium catalyst was formed in situ by the addition of chromium chloride and the oxazoline ligand **(*R*,*S*)-131**. Manganese acted as a reducing agent, and Cp_2_ZrCl_2_ as a dissociation reagent. Then, removal of the silyl group with tetrabutylammonium fluoride gave advanced intermediate **132** in 82% yield, thus accomplishing the formal synthesis of the desired **(+)-123** (Scheme 16).

## 5. Conclusions

Terpenes and related compounds represent an important family of marine-derived metabolites. The great number of studies focused on their biological activities and total synthesis remark their importance. We have summarized the most active compounds for a variety of bioactivities (see Table 2). *Trans*-oxylipid has shown nematocidal properties against *Haemonchus contortus* (LD_50_ = 1.8 ppm) and *Trichostrongyllus colubriformis* (LD_50_ = 9.9 ppm). These values, comparable to the commercial levamisole and closantel, make this compound an interesting scaffold for the development of antihelmintic substances. Though not related to drug development, we want to remark the biological interest of (+)-PMA. Its potent olfactory response in sea lampreys makes it very valuable for the control and conservation of lamprey populations. Efforts should be made in developing a total synthesis for this compound, as none has been reported to date. Mutafuran D has antifungal activity, showing a moderate value of MIC = 4 mg/mL against *Cryptococcus neoformans var. grubii*. Although not an impressive value, further studies could shed light on the structure–activity relationship of this brominated ene-yne tetrahydrofuran derivative. Furoplocamioid C could serve as a lead for novel biopesticides, as it has shown a potent antifeedant effect against *Leptinotarsa decemlineata*, with an EC_50_ of 19.1 nmol/cm^2^, *Myzus Persicae* (EC_50_ of 3.7 nmol/cm^2^), and *Ropalosiphum Padi* (EC_50_ of 1.6 nmol/cm^2^) but it has low mammalian toxicity and phytotoxic effects. With respect to antibacterial activity, heranopyrrole D has shown very good values against *Staphylococcus aureus* ATCC 25923 (IC_50_ = 1.8 mM), *Staphylococcus epidermidis* ATCC 12228 (IC_50_ = 0.9 mM), and *Bacillus subtilis* ATCC 6633 (IC_50_ = 1.8 mM); thus, the nitropyrrole moiety should be further studied to determine whether it plays an important role in its antibacterial behavior. On the other hand, though (+)-darwinolide shows a more moderate value against *Staphylococcus aureus* (IC_50_ = 33.2 μM), its selectivity towards the biofilm phase and its low toxicity makes it a very good lead for the development of antibiofilm-specific antibiotics.

Regarding antitumoral applications, there are several compounds that have shown very promising activities. Uprolide D acetate has cytotoxic activity against different tumor cell lines: HeLa cells (IC_50_ = 2.5 mM), CCRF-CEM T-cell leukemia (IC_50_ = 7.0 mM), HCT-116 colon cancer (IC_50_ = 7.0 mM), and MCF-7 breast adenocarcinoma (IC_50_ = 0.6 mM). Thyrsenol B has an impressive potential against murine lymphoid neoplasm P-388 cells (IC_50_ = 0.016 mM). Saiyacenol B shows antiproliferative activity against tumor cell lines MM144 human multiple myeloma (IC_50_ = 11.0 mM), HeLa human cervical carcinoma (IC_50_ = 24.5 mM), CADO-ES1 human Ewing’s sarcoma (IC_50_ = 14.0 mM), and possesses a very good value for Jurkat (human T-cell acute leukemia): IC_50_ = 2.7 mM. Furthermore, it presents antifouling activity against *Navicula* cf. *salinicola* (IC_50_ = 17.2 mM) and *Cylindrotheca* sp. (IC_50_ = 17.0 mM). Finally, (+)-varitriol is a very interesting example, as it is quite a simple molecule with powerful properties. It presents submicromolar activities against different cancer cell lines, namely RXF-393 (Renal cancer cell) GI_50_ = 0.16 μM; SNB-75 (CNS cancer cell) GI_50_ = 0.24 μM; and DU-145 (breast cancer cell) GI_50_ = 0.11 μM. Although some efforts have already been made and different total syntheses reported, we believe that further studies should point to the synthesis of analogs and the study of structure–activity relationships in this simple scaffold.

Regarding antiprotozoal activity, saiyacenol B shows a good IC_50_ of 10.3 mM against *Leishmania amazonensis*. Interestingly it was surpassed by its synthetic counterpart, 28-iodo-saiyacenol B, with a value of IC_50_ = 5.4 mM pointing to an interesting effect of this 28-iodo-substitution. Alisiaquinone C presents antimalarial activity in vitro against the chloroquine-resistant strains MC29 CQR (IC_50_ = 0.08 mM) and B1 CQR (IC_50_ = 0.21 mM), and the chloroquine-sensitive strain F32 CQS (IC_50_ = 0.15 mM). Though it presents a high toxicity, similar structures that preserve antimalarial activity and have reduced toxicity values could be developed. A possible lead for anti-inflammatory development is myrothecol: it shows antioxidant activity with an EC_50_ = 1.2 mg/mL (*S* isomer) and EC_50_ = 1.4 mg/mL (*R* isomer), inhibits nitric oxide formation, and displays anti-inflammatory activity. It is also a small molecule and, thus, its synthesis will require lower effort.

In conclusion, the tetrahydrofuran moiety is a common motif found in a variety of such compounds, which has meant a particular emphasis of researchers on the development of new methodologies for the synthesis of such derivatives. In this context, total synthesis is a powerful tool to have access to these natural products. Firstly, it is an invaluable means for the determination of the structure and total configuration of natural compounds since, in some cases, the available NMR methods are insufficient in definitively determining the structures of biologically relevant substances. Secondly, since extracting and purifying compounds from natural sources are difficult and time-consuming processes, total synthesis has emerged as a suitable solution for the production of larger amounts of compounds, thus bringing possibilities for further biological studies, the discovery of novel drug candidates, and the expansion of the medicinal chemistry frontiers.

## Figures and Tables

**Table 1:** Overview of compounds from this review, their sources, classes, and species (background color code coincides with that of Figure 1).

Marine Source	Compound Class	Marine Species	Compound Name
Lampreys 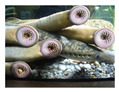	Fatty acids	*Petromyzon marinus* L.	(+)-Petromyroxol (−)-Petromyroxol (+)-PMA (−)-PMA (+)-PMB (−)-PMB
Corals 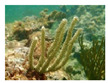	Diterpenes	*Eunicea mammosa*	Uprolides D–E
	Substituted THF	*Sinularia* sp.	Sinularones E–F
Algae 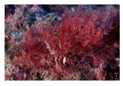	C19 lipid diols	*Notheia anomala*	*Trans*-oxylipid *Cis*-oxylipid
	Monoterpenes	*Plocamium cartilagineum*	Furoplocamioids A–C
		*Pantoneura plocamioides*	Pantofuranoids A–F
	Sesquiterpenes	*Laurencia nipponica* Yamada	(−)-Kumausallene (+)-*Trans*-Kumausyne
	Triterpenes	*Laurencia intricata*	Intricatetraol
		*Laurencia omaezakiana*	Omaezakianol
		*Laurencia viridis*	Longilenes Laurokanols A–E Yucatecone Thyrsenols A–B Saiyacenols A–C
		*Laurencia obtusa*	Teurilene
		*Laurencia thyrsifera*	Thyrsiferol
Sponges 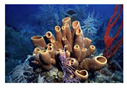	Fatty acids	*Xestospongia muta*	Mutafurans A–G
		*Xestospongia testudinaria*	Mutafuran H
		*Aspergillus* sp. LS78	Aspericacid A–B
	Diterpenes	*Dendrillamembranosa*	(+)-Darwinolide
	Triterpenes	*Rhabdastrella* sp.	Rhabdastins H–I
	Meroterpenes	New Caledonian	Alisiaquinones A–C Alisiaquinol
	Substituted THF	*Pachastrissa/Jaspis* sp.	Jaspines A-B
Fungi 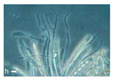	Meroterpenes	*Alternaria alternata*	Tricycloalterfurenes A–D
		*Stemphylium* sp.	Tricycloalterfurenes E–G
	Substituted THF	*Myrothecium* sp. BZO-L062	(−)-1*S*-Myrothecol (−)-1*R*-Myrothecol Methoxy-myrothecol
		*Astrosphaeriella nypae* BCC-5335	Astronypyrone Astronyquinone Astronyurea
		*Emericella variecolor*	(+)-Varitriol (−)-Varitriol
Bacteria 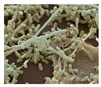	Sesquiterpenes	*Streptomyces* sp. CMB-M0423	Heronapyrroles C–D
	Meroterpenes	*Streptomyces* sp. AJS-327 and CNQ-253	Marinoterpins A–C

**Table 2:** Summary of most active compounds for a range of bioactivities within this review.

Compound	Biological Activity	Cell Line or Organism	Biological Result	Assay	Reference
*trans*-oxylipid	Nematocidal	*Haemonchus contortus*	LD_50_ = 1.8 ppm	Parasitic nematode larval development assay	[21]
		*Trichostrongylus colubriformis*	LD_50_ = 9.9 ppm		
(+)-PMA	Pheromone	*Petromyzon marinus*	10^−11^ molL^−1^	Electro-olfactogram	[26]
Mutafuran D	Antifungal activity	*Cryptococcus neoformans var. grubii*	MIC = 4 μg/mL	Pathogenic fungus assay	[27]
Furoplocamioid C	Antifeedant effects	*Leptinotarsa decemlineata*	EC_50_ = 19.1 nmol/cm^2^	Insect bioassay	[32]
		*Myzus persicae*	EC_50_ = 3.7 nmol/cm^2^		
		*Ropalosiphum padi*	EC_50_ = 1.6 nmol/cm^2^		
Heranopyrrole D	Antibacterial activity	*Staphylococcus aureus* ATCC 25923	IC_50_ = 1.8 μM	Antibacterial assay	[36]
		*Staphylococcus epidermidis* ATCC 12228	IC_50_ = 0.9 μM		
		*Bacillus subtilis* ATCC 6633	IC_50_ = 1.8 μM		
(+)-Darwinolide	Antibacterial activity	*Staphylococcus aureus*	IC_50_ = 33.2 μM	MRSA biofilm assay	[49]
Uprolide D acetate	Cytotoxic Activity Against Tumor Cell Lines	HeLa cells	IC_50_ = 2.5 µg/ m	Cytotoxicity assay on human cells	[52]
		CCRF-CEM T-cell leukemia	IC_50_ = 7.0 µg/ mL		
		HCT 116 colon cancer	IC_50_ = 7.0 µg/ mL		
		MCF-7 breast adenocarcinoma	IC_50_ = 0.6 µg/ mL		
Thyrsenol B	Cytotoxic Activity Against Tumor Cell Lines	Murine lymphoid neoplasm P-388 cells	IC_50_ = 0.016 μM	Cytotoxicity assays on human cells	[74]
	Inhibitory activity	Protein phosphatase PP2A	Inhibition > 90% ([PP2A] > 10 μM)	Enzymatic assay	[75]
Saiyacenol B	Antiproliferative activity Against Tumor Cell Lines	MM144 (human multiple myeloma) Jurkat (human T-cell acute leukemia)	IC_50_ = 11.0 μM	Cytotoxicity assays on human cells	[78]
		HeLa (human cervical carcinoma)	IC_50_ = 24.5 μM		
		CADO-ES1 (human Ewing’s sarcoma)	IC_50_ = 14.0 μM		
		Jurkat (human T-cell acute leukemia	IC_50_ = 2.7 μM		
	Antifouling activity	*Navicula* cf. *salinicola*	IC_50_ = 17.2 μM	Diatom growth inhibition	
		*Cylindrotheca* sp.	IC_50_ = 17.0 μM	Inhibition of macroalgal spore germination	
	Anti-protozoal activity	*Leishmania amazonensis*	IC_50_ = 10.3 μM	In vitro susceptibility assay	[80]
28-iodo-saiyacenol B	Anti-protozoal activity	*Leishmania amazonensis*	IC_50_ = 5.4 μM	In vitro susceptibility assay	[80]
Alisiaquinone C	Antimalarial activity (in vitro)	MC29 CQR	IC_50_ = 0.08 μM	[^3^H]- Hypoxanthine incorporation	[90]
		B1 CQR	IC_50_ = 0.21 μM		
		F32 CQS	IC_50_ = 0.15 μM		
	Antimalarial activity (in vivo)	Rodent malaria	Mortality day 5 = 0 (5 mg/kg/day) Mortality day 5 = 80 (20 mg/kg/day)	four-day suppressive in vivo assay	
(−)-1*S*-Myrothecol	Antioxidant activity		EC_50_ = 1.2 µg/mL	ABTS assay	[100]
			μM Trolox Equiv/μM = 1.4	ORAC assay	
(+)-1*R*-Myrothecol	Antioxidant activity		EC_50_ = 1.4 µg/mL	ABTS assay	
			μM Trolox Equiv/μM = 1.2	ORAC assay	
(+)-Varitriol	Cytotoxic activity to cancer cell lines	RXF-393 (Renal cancer cell)	GI_50_ = 0.16 μM	Cytotoxicity assays	[104]
		SNB-75 (CNS cancer cell)	GI_50_ = 0.24 μM	Cytotoxicity assays	
		DU-145 (breast cancer cell)	GI_50_ = 0.11 μM	Cytotoxicity assays	

## Data Availability

Data sharing not applicable.

## Abbreviations

Ac: acetate; Aq.: aqueous; BDSB: bromodiethylsulfonium bromopentachloroantimonate; Bn: benzyl; Boc: tert-butyloxy carbonyl; Bu: butyl; Bz: benzoyl; Cat.: catalyst; CD: circular dichroism; Cp: cyclopentadienyl; CSA: camphorsulfonic acid; Cy: cyclohexyl; D-(−)-DET: (−)-diethyl D-tartrate; DDQ: 2,3-Dichloro-5,6-dicyano-1,4-benzoquinone; DFT: density functional theory; (DHQD)_2_PYR: hydroquinidine-2,5-diphenyl-4,6-pyrimidinediyl diether; DIAD: diisopropyl azodicarboxylate; DME: dimethoxyethane; DMSO: dimethylsulfoxide; *dr*: diastereomeric ratio; ECD: electronic circular dichroism; *ee*: enantiomeric excess; HMDS: hexamethyl disilazide; HPLC: high-performance liquid chromatography; IBX: orto-iodoxybenzoic acid; IR: infrared; M: molar; MS: mass spectrometry; Ms: methanesulfonyl; NMR: nuclear magnetic resonance; PAB: para-aminobenzoate; PG: protecting group; Ph: phenyl; PMA: petromyric acid A; PMB: petromyric acid B or *para*-methoxybenzyl; PNB: *para*-nitrobenzoate; *p*-NBA: *para*-nitrobenzoic acid; Pr: propyl; TBAF: tetrabutylammonium fluoride; TBHP: tert-butyl hydroperoxide; TIPS: triisopropylsilyl; TfOH: trifluoromethanesulfonic acid; TMS: trimethylsilyl.
